# Comparison of the efficacy of administering a combination of ezetimibe plus fenofibrate versus atorvastatin monotherapy in the treatment of dyslipidemia

**DOI:** 10.1186/1476-511X-8-56

**Published:** 2009-12-17

**Authors:** Shoba Sujana Kumar, Karen A Lahey, Andrew Day, Stephen A LaHaye

**Affiliations:** 1 University of Toronto, Division of Endocrinology & Metabolism, Toronto, Ontario, Canada; 2 Women's College Hospital, Department of Medicine and Division of Endocrinology, Toronto, Ontario, Canada; 3 Hotel Dieu Hospital, Kingston, Ontario, Canada; 4 Kingston General Hospital, Kingston, Ontario, Canada; 5 Queen's University, Department of Medicine and Division of Cardiology, Kingston, Ontario, Canada

## Abstract

**Background:**

This trial compares the efficacy of administering a combination of ezetimibe plus fenofibrate as an alternative to statin monotherapy for the treatment of dyslipidemia. In this randomized, unblinded crossover study, 43 patients with documented hypercholesterolemia requiring pharmacotherapy were randomized to receive six weeks of either a combination of 10 mg of ezetimibe plus 160 mg of fenofibrate (combination) or 10 mg of atorvastatin monotherapy (atorvastatin). The primary endpoint was the percentage reduction of low-density lipoprotein cholesterol (LDL-C).

**Results:**

LDL-C decreased by 34.6% with the combination therapy versus 36.7% with atorvastatin monotherapy. The difference between the two groups was not statistically significant (p = 0.46). Both study interventions provided similar improvements in total cholesterol (-25.1% with combination versus -24.6% with atorvastatin, p = 0.806) and high-density lipoproteins (+10.0% with combination versus +8.9% with atorvastatin, p = 0.778). Combination therapy showed a trend towards a greater reduction in triglycerides (-25.4% with combination versus -14.5% with atorvastatin, p = 0.079), although there was no significant difference between the two study interventions in terms of the improvement in the TC:HDL ratio (-29.0% with combination versus -28.7% with atorvastatin, p = 0.904).

**Conclusions:**

The combination of ezetimibe plus fenofibrate appeared to produce nearly identical alterations in serum lipoprotein levels when compared to monotherapy with 10 mg of atorvastatin. Daily treatment with the combination of ezetimibe plus fenofibrate is an acceptable alternative to atorvastatin for the treatment of dyslipidemia in patients who are intolerant of statins.

## Background

3-hydroxy-3-methylgluatryl-coenzyme A reductase inhibitors (statins) are the most potent and frequently used drugs for the treatment of hypercholesterolemia. Statin therapy has been shown to reduce the rate of major vascular events in patients with established vascular disease [1], and is considered the first line therapy for the management of dyslipidemia in such individuals [2]. The withdrawal of cerivastatin from the U.S. market on August 8, 2001, however, has prompted concern on the part of physicians and patients regarding the safety of statins. Although statins are well tolerated by the majority of patients, known side effects include transaminitis, myalgias, myositis and very rarely, rhabdomyolysis [3]. Whether perceived or real, concerns over these potential side effects can result in the discontinuation of statin therapy in a significant minority of patients [4]. Until recently, however, there have not been any safe, effective and well-tolerated alternatives to statin therapy for the management of dyslipidemia in patients with vascular disease.

Ezetimibe is a novel cholesterol absorption inhibitor that prevents cholesterol absorption by inhibiting the transport of cholesterol across the intestinal wall. Treatment with ezetimibe as monotherapy results in a reduction of LDL cholesterol (LDL-C) by approximately 18% [5], and has been shown to be well tolerated, with a safety profile similar to that of placebo [6,7]. When compared to the effect of statins on the lowering of LDL-C, however, ezetimibe would appear to provide only a modest benefit [8].

Fenofibrate is a fibric acid derivative that binds to peroxisome proliferator-activated receptor alpha and alters lipoprotein synthesis [9]. Treatment with fenofibrate monotherapy has also been proven to provide modest reductions in LDL-C, and may also be an effective therapeutic option for patients who are intolerant of statins [10]. And while both ezetimibe and fenofibrate each provide only modest reductions in LDL-C when used as monotherapy, the co-administration of ezetimibe with fenofibrate has been shown to produce significantly greater reductions in LDL-C than with either drug alone, suggesting that the combination may be an effective second-line therapeutic option in patients who are intolerant of statins [9]. The safety of the combination has been shown both in short and long-term trials. Farnier et al. in 2005 demonstrated the safety and efficacy of the combination in a cohort with mixed hyperlipidemia over a twelve-week period [11]. More recently, McKenney et al. demonstrated that the combination of fenofibrate and ezetimibe, delivered over 48 weeks, was both well-tolerated and efficacious, in comparison with fenofibrate alone [12].

To date, we are not aware of any study which has compared the effectiveness and tolerability of the co-administration of ezetimibe and fenofibrate with statin therapy in patients with hypercholesterolemia. Accordingly, this study was designed to test the hypothesis that the combination of ezetimibe and fenofibrate is an effective alternative to statin use for statin-intolerant patients with hypercholesterolemia and vascular disease.

## Results

### Study design

**Figure 1:**
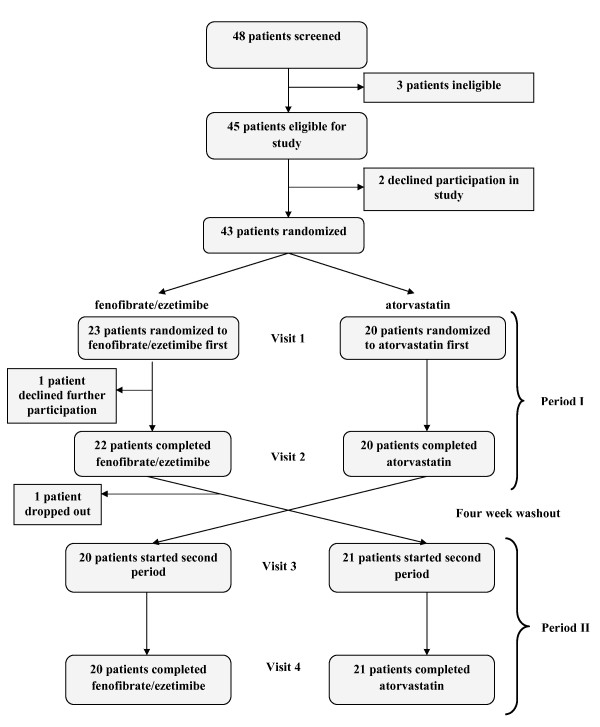
**Enrollment and Outcomes**.

Between January 2005 and February 2006, 48 patients were evaluated for eligibility and 45 patients deemed eligible (Figure 1). Two patients withdrew prior to randomization. Twenty-three patients were initially randomized to start with the combination and twenty patients were randomized to initiate therapy with atorvastatin. A further two patients subsequently declined further participation in the study; both were from the combination group. 41 patients therefore completed the study; 21 in the initial combination group and 20 in the initial atorvastatin group. The reasons for early discontinuation of the study were primarily withdrawal of consent and subsequent loss to follow-up. The baseline demographic and laboratory characteristics are listed in Table 1. The two groups were similar with respect to all baseline characteristics except for age - the patients who began the study with the combination therapy group were somewhat younger than those who began the study with atorvastatin alone. Nearly two-thirds of all patients had been on prior lipid-lowering therapy, all with statins, which were discontinued prior to the study washout period.

**Table 1:** Baseline Demographics Mean (SD) or %

	Atorvastatin	Combination
Age (years)	67.09 (9.668)	60.50 (9.561)
Male Sex	85	73.9
		
Coronary artery disease (%)	60	43.5
Angina	40	39.1
Previous Myocardial Infarction	35	13
Previous PCI	40	21.7
Previous CABG	15	21.7
Cerebrovascular Disease (%)	45	60.9
Peripheral Vascular Disease (%)	0	4.3
		
Lipid Parameters		
Total Cholesterol (mmol/L)	5.69 (0.78)	5.98 (0.91)
Low density lipoprotein (mmol/L)	3.58 (0.53)	3.91 (0.71)
High density lipoprotein (mmol/L)	1.31 (0.28)	1.44 (0.20)
Triglycerides (mmol/L)	1.75 (0.70)	1.35 (0.49)
TC/HDL Ratio	4.49 (0.97)	4.20 (0.65)
		
Smoking Status (%)		
Never Smoked	25	34.8
Previous Smoker	70	52.2
Current Smoker	5	13
		
Diabetes Mellitus (%)	10	30.4
Body Mass Index (kg/m^2^)	27.8 (3.50)	28.2 (5.43)
Weight (kg)	83.2 (12.80)	82.2 (17.82)
		
History of Hypertension (%)	60	65.2
Systolic Blood Pressure (mmHg)	128.4 (16.14)	121.4 (15.88)
Diastolic Blood Pressure (mmHg)	73.2 (8.57)	72.9 (11.68)
Mean hsCRP	3.62 (3.59)	2.91 (4.18)
		
Antithrombotic Therapy # (%)	19 (95)	23 (100)
Previous Lipid-Lowering Therapy # (%)	12 (60)	15 (65.2)
		
ACE-I/ATII Therapy # (%)	15 (75.0)	20 (87.0)

### Lipid profile effects

**Figure 2:**
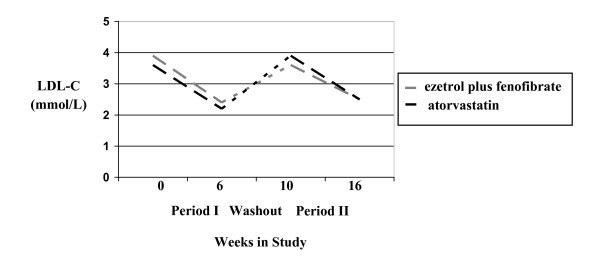
**Comparison of Ezetimibe Plus Fenofibrate Versus Atorvastatin On LDL-C Reduction Over Time**.

During the six weeks of treatment, LDL-C decreased by 34.6% (p < 0.001) with the combination therapy versus 36.7% (p < 0.001) with atorvastatin monotherapy (Figure 2 and Table 2). The difference between the two interventions was not statistically significant (2.1 +/- 2.8%, p = 0.46). Both study interventions provided similar improvements in TC (-25.1%, p < 0.001 with combination therapy versus -24.6%, p < 0.001 with atorvastatin monotherapy, p = 0.806 for difference between the two groups) and HDL-C (10.0%, p = 0.002 with combination therapy versus 8.9%, p = 0.006 with atorvastatin monotherapy, p = 0.778 for difference between the two groups). Combination therapy showed a non-significant *trend *towards a greater reduction in triglycerides (-25.4%, p < 0.001 with combination therapy versus -14.5%, p = 0.002 with atorvastatin monotherapy, p = 0.079 for difference between the two groups), though there was no significant difference between the two study interventions in terms of the improvement in the TC:HDL ratio (-29.0%, p < 0.001 with combination therapy versus -28.7%, p < 0.001 with atorvastatin monotherapy, p = 0.904 for difference between the two groups).

**Table 2:** Baseline and Changes from Baseline in Total Cholesterol, LDL-C, HDL-C, Triglycerides, TC:HDL Ratio, Apo A and Apo B after 6 Weeks of Treatment with either the Combination of Ezetimibe plus Fenofibrate or Atorvastatin Monotherapy

	Ezetimibe plus Fenofibrate				Atorvastatin Monotherapy					
	Baseline	6 Weeks	% Change	p Value	Baseline	6 Weeks	% Change	p Value	% Difference	p Value
Total Chol (mmol/L)	5.9	4.4	-25.1	< 0.001	5.8	4.4	-24.6	< 0.001	-0.4	0.806
LDL-C (mmol/L)	3.8	2.4	-34.6	< 0.001	3.7	2.3	-36.7	< 0.001	2.1	0.46
HDL-C (mmol/L)	1.4	1.5	10.0	0.002	1.3	1.5	8.9	0.006	1.1	0.778
Triglycerides (mmol/L)	1.5	1.0	-25.4	< 0.001	1.6	1.4	-14.5	0.002	-11.0	0.079
TC:HDL Ratio	4.3	3.0	-29.0	< 0.001	4.4	3.1	-28.7	< 0.001	-0.2	0.904
										
Apo A	1.27	1.32	4.5	0.041	1.29	1.30	1.5	0.493	3.04	0.295
Apo B	1.21	0.82	-31.8	< 0.001	1.19	0.82	-29.8	< 0.001	-1.99	0.392

The apolipoprotein A and B levels before and after each six-week treatment period were also examined. While there was no significant change in apolipoprotein A levels with atorvastatin monotherapy (+1.5%, p = 0.493), there was a small, albeit statistically significant increase in apolipoprotein A levels in the combination therapy group (+4.5%, p = 0.041). There was, however, no significant difference on apolipoprotein A levels (3.04 +/- 2.9%, p = 0.295) between the two treatment groups. Apolipoprotein B levels declined in both treatment groups (-31.8%, p < 0.001 with combination therapy versus -29.8%, p < 0.001 with atorvastatin monotherapy). There was no significant difference in change of apolipoprotein B level between the two groups (-1.99 +/- 2.3%, p = 0.392).

### Other results

The level of hsCRP did not show significant change with either combination therapy or atorvastatin monotherapy. In the combination therapy group there was a non-significant decrease in hsCRP of 0.1 mmol/L (range of -1.0 to 0.5, p = 0.49). The median change in hsCRP in the atorvastatin monotherapy group was a decrease of 0.1 mmol/L with a range from -0.8 mmol/L to 0.8 mmol/L of change, with p = 0.75. As a result, there was no significant difference in the change in hsCRP between the two groups (decrease of 0.1 mmol/L overall, with range -1.5 to 1.2, p = 0.99).

### Adverse events

No serious adverse events occurred during the study. One patient, who was randomized to begin with the combination therapy, noted dizziness and headache while on combination therapy and declined further participation after the first phase of the study (i.e. after completion of the combination therapy). Two patients complained of constipation while on combination therapy, whereas one complained of constipation while on atorvastatin monotherapy. Two patients complained of abdominal cramping while on combination therapy, and one of these patients stopped the combination therapy twelve days early as a result. Two patients complained of dizziness and postural changes while on atorvastatin monotherapy, but these were felt to be secondary to ACE inhibitor use and resolved with dosage adjustment of the ACE inhibitor.

None of the patients in this study developed elevations in serum aminotransferase levels (alanine aminotransferase [ALT] or aspartate aminotransferase [AST]), as defined by an aminotransferase elevation >3 times the upper limit of normal [13] at any time throughout the duration of the study. None of the patients experienced symptomatic myopathy, as defined by complaints referable to skeletal muscle, including myalgias (muscle pain), weakness (by complaint or objective testing) and cramps [14], at any time throughout the duration of the study. However, there were 23 instances of asymptomatic myopathy (as defined by CK elevations without symptoms or objective evidence of weakness [14]) in 14 subjects that were associated with mild (as defined by CK levels greater than normal, but less than 10 times the upper limit of normal [14]) CK elevation. Seven of these instances occurred while subjects were not taking any antidyslipidemic medication (i.e. they were in the washout phase). Seven instances occurred while subjects were taking combination therapy, and nine instances occurred while subjects were taking atorvastatin monotherapy. There were no instances of asymptomatic myopathy that were associated with moderate (as defined by CK levels equal to or greater than 10 times the upper limit of normal but less than 50 times the upper limit of normal [14]) or severe (as defined by CK levels equal to or greater than 50 times the upper limit of normal [14]) CK elevation.

## Discussion

The combination of ezetimibe plus fenofibrate in this study produced nearly identical alterations in serum lipoprotein levels when compared to monotherapy with 10 mg of atorvastatin. Combination therapy with ezetimibe plus fenofibrate did produce small, albeit statistically significant increases in apolipoprotein A levels, while none were observed with atorvastatin monotherapy. While both combination therapy and atorvastatin monotherapy significantly decreased triglycerides levels, combination therapy showed a trend towards a greater reduction in triglycerides than with atorvastatin monotherapy. Both treatments were generally well tolerated and there were no clinically important increases in either transaminitis or myopathy.

It is important, however, to emphasize that atorvastatin was delivered at a low dose in our study, and that one would expect more significant effects on TC, LDL-C and TC:HDL ratio with the utilization of higher doses of atorvastatin, as is the case in the contemporary management of dyslipidemia in patients with cardiovascular disease [2]. In contrast, the doses of fenofibrate and ezetimibe used in this study are the maximum approved doses of these agents and further titration is not possible.

Moreover, in contrast to statin therapy, which has an overwhelming burden of evidence to support its efficacy for the prevention of cardiovascular events, neither ezetimibe [15], nor fenofibrate [16], either alone or in combination has been shown to produce similar reductions in cardiovascular morbidity or mortality. Accordingly statin therapy is, and should remain, first line therapy for the management of dyslipidemia in patients with cardiovascular disease despite of the results of this study. Further, statin therapy, because it requires only a single daily pill, may be associated with higher degrees of patient adherence to therapy than combination therapy. Combination therapies are also more expensive than monotherapies - cost may therefore also limit the utility of the combination therapy outside of the clinical trial setting.

Nevertheless, a significant minority of patients discontinue statin therapy due to concerns over side effects, whether these concerns are perceived or real. For instance, in the PRIMO Study [4], between 5% and 18% of patients experienced muscular symptoms while receiving high-dosage statin therapy in an outpatient setting. As a consequence, many of these patients may elect to discontinue treatment with statin medication, and for them, an alternative to statin therapy would be useful, despite the aforementioned advantages of statin monotherapy.

Based upon the results of the present study, the substitution of atorvastatin with the combination of ezetimibe and fenofibrate would seem to be a reasonable option in such patients who are intolerant of statin therapy, recognizing that the combination of ezetimibe plus fenofibrate has not been determined to prevent cardiovascular disease in randomized controlled trials. For instance, in the ENHANCE Trial, treatment with ezetimibe as monotherapy resulted in a reduction of LDL cholesterol (LDL-C) by 16.5% [15], but did not significantly reduce progression of carotid atherosclerosis, as determined by measurement of carotid intimal medial thickening. In the FIELD Study, treatment with fenofibrate as monotherapy resulted in a reduction of LDL cholesterol (LDL-C) by approximately 14.7% compared to those patients who did not start other lipid-lowering therapy [16], but did not significantly reduce the risk of coronary events.

Whether a 15-20% reduction in LDL-C is enough to significantly reduce the risk of cardiovascular also remains to be seen. For instance, in the ALLHAT-LLT Trial, no significant difference in all-cause mortality or combined fatal and nonfatal CHD was demonstrated between the pravastatin and the usual care groups [17]. This was in the context of a 16.7% differential in LDL-C between the two treatment groups.

And while more modest reductions in LDL-C with either ezetimibe or fenofibrate monotherapy may not be sufficient to realize clinically important reductions in cardiovascular outcomes, the more aggressive approach of combining ezetimibe and fenofibrate may be more effective in achieving LDL-C lowering and clinically important and statistically significant differences in cardiovascular outcomes. Based on the data from the meta-analysis, which was performed by the Cholesterol Treatment Trialists' (CTC) Collaborators, a reduction of LDL-C by the 1.4 mmol/L that was achieved with the combination of ezetimibe plus fenofibrate would result in a reduction in major coronary events by 32% [18]. As such, the combination may have the potential to realize similar results to the Heart Protection Study [19], which achieved a significant 24% reduction in major adverse cardiac events with a 29% reduction in LDL-C.

### Limitations

This study was not blinded or powered to detect differences in clinical outcomes between the two treatment groups. Prior use of study medications and the short duration of therapy in this study may also have minimized potential safety and tolerability issues - six weeks on each therapy may not have been enough time to detect significant differences in adverse effects of each medication. Accordingly, the consequences of long-term use of combination therapy with ezetimibe plus fenofibrate are not known and would require the completion of larger, randomised and blinded studies.

## Conclusions

The combination of ezetimibe 10 mg daily plus fenofibrate 160 mg daily appeared to produce nearly identical alterations in serum lipoprotein levels when compared to monotherapy with 10 mg of atorvastatin. Daily treatment with the combination of ezetimibe plus fenofibrate is an acceptable alternative to atorvastatin for the treatment of dyslipidemia in patients who are intolerant of statins.

## Methods

### Research design

Men and women between the ages of 18 and 85 were recruited from the Southeastern Ontario Vascular Disease Prevention and Research Centre, located at Queen's University in Kingston, Ontario. Patients were eligible for the study if they had documented hypercholesterolemia, with an LDL-C≥3.0 mmol/L. Patients were excluded if they had a history of hepatic disease (AST or ALT ≥ 3 ULN), elevation of creatine kinase (CK ≥ 3 ULN), uncontrolled diabetes (HbA1c ≥ 10%), intolerance or hypersensitivity to statins, fibric acid derivatives or ezetimibe, were pregnant or breastfeeding, had a history of excessive alcohol use, were enrolled in another study, or had an acute coronary syndrome or stroke within 6 weeks of enrolment. All patients provided written informed consent. This study was approved by the Research Ethics Board at Queen's University in Kingston, Ontario.

The study design was that of an unblinded but randomized cross-over study. Patients who were previously on lipid-lowering therapy underwent a 4-week washout period before entry into the study. Prior to randomization, height, weight, heart rate, and blood pressure were recorded. Blood work was drawn after a 14 hour fast, and analyzed for the following constituents: lipid profile (total cholesterol (TC), low-density lipoprotein cholesterol (LDL-C), high density lipoprotein cholesterol (HDL-C), triglycerides (TG), total cholesterol to high density lipoprotein ratio (TC/HDL), apolipoprotein A and apolipoprotein B), high sensitivity C-reactive protein (hsCRP), aspartate aminotransferase (AST), alanine aminotransferase (ALT), alkaline phosphatase (ALP), bilirubin, creatine kinase (CK), fasting glucose and HbA1c (for patients with diabetes), electrolytes, and creatinine. Total cholesterol and triglyceride concentrations were measured enyzmatically. All enzymatic lipid testing was performed on the Roche Modular (Roche Diagnostics Canada, Canada). LDL-C was calculated using the Friedewald formula applied to the values measured.

Patients were then randomly assigned to receive either a combination of 10 mg of ezetimibe plus 160 mg of fenofibrate (combination) or 10 mg of atorvastatin monotherapy (atorvastatin) for 6 weeks. Patients were not blinded to treatment allocation. After completion of the first phase of the study, patients underwent a 4-week washout period. The patients were then switched to the other regimen for another 6 weeks. Following this phase, patients had their involvement in the study concluded, and were placed back on statin therapy for their hypercholesterolemia, and post-study usual follow-up was arranged with their regular physicians. After the end of each treatment regimen and washout period, patients returned to clinic for repeat measurements and blood work. At each visit, data related to lipid profile, compliance, and side effects were recorded. In total, patients came for 4 visits, including visits at baseline, six, ten, and sixteen weeks.

### Statistical analysis

The hypothesis was that a combination of ezetimibe and fenofibrate would provide equal lowering of LDL-C in comparison with atorvastatin at low dose. With this in mind, an absolute acceptable difference between the two groups was deemed to be a 20% difference in LDL-C reduction between the two groups. With a significance of 0.05 and a power of 80%, the calculation was therefore made that 44 patients would be required for this study; twenty-two patients would be in each arm of the study initially, and would cross-over to the other group at the half-way mark.

The data from the study was entered into a Microsoft Excel spreadsheet as it was obtained, and subsequently imported into SPSS for Windows for statistical analysis. Paired t-tests using a 95% confidence interval were used to compare the concentrations of total cholesterol, LDL-C, HDL-C, triglycerides and hsCRP between the two groups.

The primary outcome measure was the difference in the percent change in LDL cholesterol over six weeks of therapy in each group. A linear mixed effects model was used to pool results over the periods of the study and estimate the average difference between the combination and atorvastatin groups after adjusting for period effects. The mixed model permitted incorporation of data from subjects with only one period (arm of study) but provides estimates identical to the classical analysis of the AB/BA crossover study as described by Fleiss and others [20-23]. The model is efficient and unbiased but assumes no differential carryover effect. Secondary endpoints included HDL-C, triglyceride, and hsCRP levels. The linear mixed-effects model was used to compare all outcomes except for hsCRP, which was compared by the non-parametric Wilcoxon Signed-Rank test.

Further analysis was performed on serum samples in an outside laboratory to compare apolipoprotein A and B levels after each washout period and after each six-week treatment. Liver enzyme levels were also drawn at each point and analyzed by the linear mixed-effects model.

All subjects with data for at least one period were analyzed, according to the intent-to-treat principle. All tests were two-sided and analyses were performed in SAS version 8.2 [23].

## List of Abbreviations Used

ACE: angiotensin-converting enzyme; ALLHAT: Anti-hypertensive and Lipid-lowering Treatment to Prevent Heart Attack Trial; ALT: alanine aminotransferase; AST: aspartate aminotransferase; CK: creatine kinase; CTC: Cholesterol Treatment Trialists' Collaboration; ENHANCE: Ezetimibe and Simvastatin in Hypercholesterolemia Enhances Atherosclerosis Regression Trial; FIELD: Fenofibrate Intervention and Event Lowering in Diabetes Study; HbA1c: Hemoglobin A1c; HDL-C: high-density lipoprotein cholesterol; hsCRP: high sensitivity C-reactive protein; LDL-C: low density lipoprotein cholesterol; PRIMO: Prediction of Muscular Risk in Observational conditions Study; TC: total cholesterol; TC:HDL: total cholesterol: HDL ratio; TG: triglycerides.

## Competing interests

The authors declare that they have no competing interests.

## Authors' contributions

SS collected all trial data and collaborated with AD on the analysis, wrote the initial draft of the manuscript and was involved in each of its revisions. KAL performed each of the study visits with patients and recorded all study data during each visit of the trial. AD performed the statistical analysis. SAL recruited patients, supervised the project, and revised the manuscript. The final manuscript was read and reviewed by SS, KAL, AD and SAL.
